# The Large Nonstructural Protein (NS1) of Human Bocavirus 1 Directly Interacts with Ku70, Which Plays an Important Role in Virus Replication in Human Airway Epithelia

**DOI:** 10.1128/JVI.01840-21

**Published:** 2022-02-23

**Authors:** Liting Shao, Kang Ning, Jianke Wang, Fang Cheng, Shengqi Wang, Jianming Qiu

**Affiliations:** a Beijing Institute of Radiation Medicine, Beijing, China; b Department of Microbiology, Molecular Genetics and Immunology, University of Kansas Medical Center, Kansas City, Kansas, USA; University of Illinois at Urbana Champaign

**Keywords:** DNA damage, DNA replication, human airway epithelia, parvovirus

## Abstract

Human bocavirus 1 (HBoV1), an autonomous human parvovirus, causes acute respiratory tract infections in young children. HBoV1 infects well-differentiated (polarized) human airway epithelium cultured at an air-liquid interface (HAE-ALI). HBoV1 expresses a large nonstructural protein, NS1, that is essential for viral DNA replication. HBoV1 infection of polarized human airway epithelial cells induces a DNA damage response (DDR) that is critical to viral DNA replication involving DNA repair with error-free Y-family DNA polymerases. HBoV1 NS1 or the isoform NS1-70 *per se* induces a DDR. In this study, using the second-generation proximity-dependent biotin identification (BioID2) approach, we identified that Ku70 is associated with the NS1-BioID2 pulldown complex through a direct interaction with NS1. Biolayer interferometry (BLI) assay determined a high binding affinity of NS1 with Ku70, which has an equilibrium dissociation constant (*K_D_*) value of 0.16 μM and processes the strongest interaction at the C-terminal domain. The association of Ku70 with NS1 was also revealed during HBoV1 infection of HAE-ALI. Knockdown of Ku70 and overexpression of the C-terminal domain of Ku70 significantly decreased HBoV1 replication in HAE-ALI. Thus, our study provides, for the first time, a direct interaction of parvovirus large nonstructural protein NS1 with Ku70.

**IMPORTANCE** Parvovirus infection induces a DNA damage response (DDR) that plays a pivotal role in viral DNA replication. The DDR includes activation of ATM (ataxia telangiectasia mutated), ATR (ATM- and RAD3-related), and DNA-PKcs (DNA-dependent protein kinase catalytic subunit). The large nonstructural protein (NS1) often plays a role in the induction of DDR; however, how the DDR is induced during parvovirus infection or simply by the NS1 is not well studied. Activation of DNA-PKcs has been shown as one of the key DDR pathways in DNA replication of HBoV1. We identified that HBoV1 NS1 directly interacts with Ku70, but not Ku80, of the Ku70/Ku80 heterodimer at high affinity. This interaction is also important for HBoV1 replication in HAE-ALI. We propose that the interaction of NS1 with Ku70 recruits the Ku70/Ku80 complex to the viral DNA replication center, which activates DNA-PKcs and facilitates viral DNA replication.

## INTRODUCTION

Human bocavirus 1 (HBoV1) was first identified in nasopharyngeal specimens of children with respiratory illness in 2005 (1). It is a member of *Primate bocaparvovirus 1* in the genus *Bocaparvovirus* of the *Parvoviridae* family (1, 2). HBoV1 is a bona fide emerging human-pathogenic respiratory virus that causes lower respiratory tract infections in young children worldwide (3–23). In *in vitro* cell culture, HBoV1 infects well-differentiated/polarized primary or immortalized human airway epithelium (HAE) cultured at an air-liquid interface (HAE-ALI) but not monolayer cultured proliferating airway epithelial cells or other types of cells (24–32). The full-length duplex genome of HBoV1 replicates in human embryonic kidney 293 cells (HEK293) and generates progeny virions that are highly infectious to HAE-ALI cultures (25–27).

Both HBoV1 infection of mitotically quiescent/nondividing human airway epithelial cells and HBoV1 DNA replication of dividing HEK293 cells initiate a DNA damage response (DDR). The DDR involves activation of all three phosphatidylinositol 3-kinase-related kinases (PI3KKs): ATM (ataxia telangiectasia mutated), ATR (ATM- and RAD3-related), and DNA-PKcs (DNA-dependent protein kinase catalytic subunit). Infection of different parvoviruses induces a DDR with activation of different PI3KKs. Protoparvovirus minute virus of mice (MVM) infection activates the ATM pathway (33, 34), ATR (34), and DNA-PKcs with its accessory proteins, i.e., the Ku70/Ku80 heterodimer, which accumulate in the virus replication centers (33), depending on the cell types. However, only ATM activation plays a role in viral DNA replication in MVM-infected A9 cells (33). In contrast, erythroparvovirus parvovirus B19 (B19V) infection of human erythroid progenitor cells, in which three major PIKK cascades are activated, leads to recruitment of the PI3KKs and their downstream transducers, Chk2, Chk1, and Ku70/Ku80, to the viral DNA replication center (35). ATR and DNA-PK signaling have been implicated as playing a role in B19V viral DNA synthesis. Bocaparvovirus minute virus of canines (MVC) infection results in activation of both ATM and ATR cascades but not DNA-PKcs, and only ATM activation is required for efficient viral replication and progeny production (36). Importantly, activation of all three PI3KKs is required for replication of the HBoV1 genome in both the dividing and nondividing cells (29). It is different from the DDR induced by other parvoviruses, in which infection only activates one or two of the PI3KKs and induces cell cycle arrest (37, 38). In contrast to HBoV1, all other known autonomous parvoviruses rely on activity of the cellular DNA replication machinery for their replication during the S phase, which is arrested during virus infection (39–45).

Parvoviruses replicate in distinct subnuclear compartments of the nucleus, the viral DNA replication centers, where essential viral DNA replication cellular factors are accumulated with the necessary viral large nonstructural protein, NS1, in autonomous parvovirus and Rep78/68 in dependoparvovirus (adeno-associated virus, AAV). During replication of autonomous parvovirus, e.g., MVM, B19V, MVC, and HBoV1, the viral DNA replication centers colocalize with the DNA damage foci induced upon virus infection (35, 42, 46). Phosphorylated DNA damage factors, including ATM, ATR, DNA-PKcs, H2AX, and RPA32, as well as the cellular replication factors, including DNA polymerase (Pol) δ, minichromosome maintenance protein complex (MCM), replication factor C (RFC), and replication protein A (RPA), are often colocalized within the viral DNA replication/DNA damage foci (35, 46).

Similarly, during productive infection of the dependoparvovirus, AAV2 DNA is recruited into the helper virus, adenovirus (AdV), herpesvirus (HSV), or DNA replication centers and takes advantage of the helper gene function, as well as cellular DNA replication factors, to promote AAV2 DNA replication (47–49). During coinfection with AdV, AAV2 infection induces a DNA-PK-dependent DDR that is localized to the AAV2 DNA replication centers (50). The Rep78/68 itself in the absence of viral infection triggers DNA-PK-dependent DDR signals (50, 51). However, the DDR signals get amplified when AAV2 replicates in the presence of helper functions, suggesting that the replicating viral genome facilitates the DDR pathway in a feedback manner (50).

Recently, a high-throughput viral chromosome conformation capture sequencing (V3Cseq) assay was established to reveal the direct interactions of a lytic virus genome with distinct regions of the cellular chromosome in genome-wide fashion (52). They identified that MVM interacts with cellular DNA damage foci to establish and amplify its lytic infection (53). These foci contain DNA replication and gene expression factors that help support viral infection (33, 54). Moreover, MVM NS1 facilitates the localization of the MVM genome to the DNA damage foci (55). Notably, it was found that the AAV2 genome and Rep78/68 also interact with cellular DNA damage sites (56). However, how NS1 or Rep78/68 is recruited to the DNA damage foci is currently unknown.

Similar to AAV2 Rep78/68, the HBoV1 counterpart NS1-100/70 *per se* induces a DDR (57). The HBoV1 NS1-induced DDR is similar to that induced during virus infection and viral DNA replication, in which all three PI3KKs are activated with phosphorylation of RPA32 and H2AX. We wondered whether NS1 interacts with some components of the DDR machinery for the inactivation of the PI3KKs, which are typically activated by damaged DNA (58, 59).

In this study, we used HBoV1 NS1 as bait to look for cellular DNA damage factors that interact with NS1 using the second-generation proximity-dependent biotin identification (BioID2) method. We identified 13 DNA damage factors that are biotinylated by the NS1-BioID2, 8 of which were confirmed to interact in a coimmunoprecipitation (co-IP) assay, including Ku70 and DNA-PKcs. Ku70 was proven to directly interact with NS1 through all three function domains, with the highest affinity at the C-terminal domain. Knockdown of Ku70 in both HAE-ALI and HEK293 cells proved the essential role of Ku70 during HBoV1 replication.

## RESULTS

### Identification of host proteins interacting with NS1.

As NS1 is indispensable in HBoV1 DNA replication (25, 29), we speculated that there are host proteins that assist NS1 in viral DNA replication. Here, we used the second-generation proximity-dependent biotin identification method (BioID2) to initially screen NS1-interacting host proteins. BioID2 uses a substantially smaller biotin ligase from Aquifex aeolicus within a residue-mutated catalytic domain (R40G) that enhances the efficiency in identification of protein-protein association (60).

**FIG 1:**
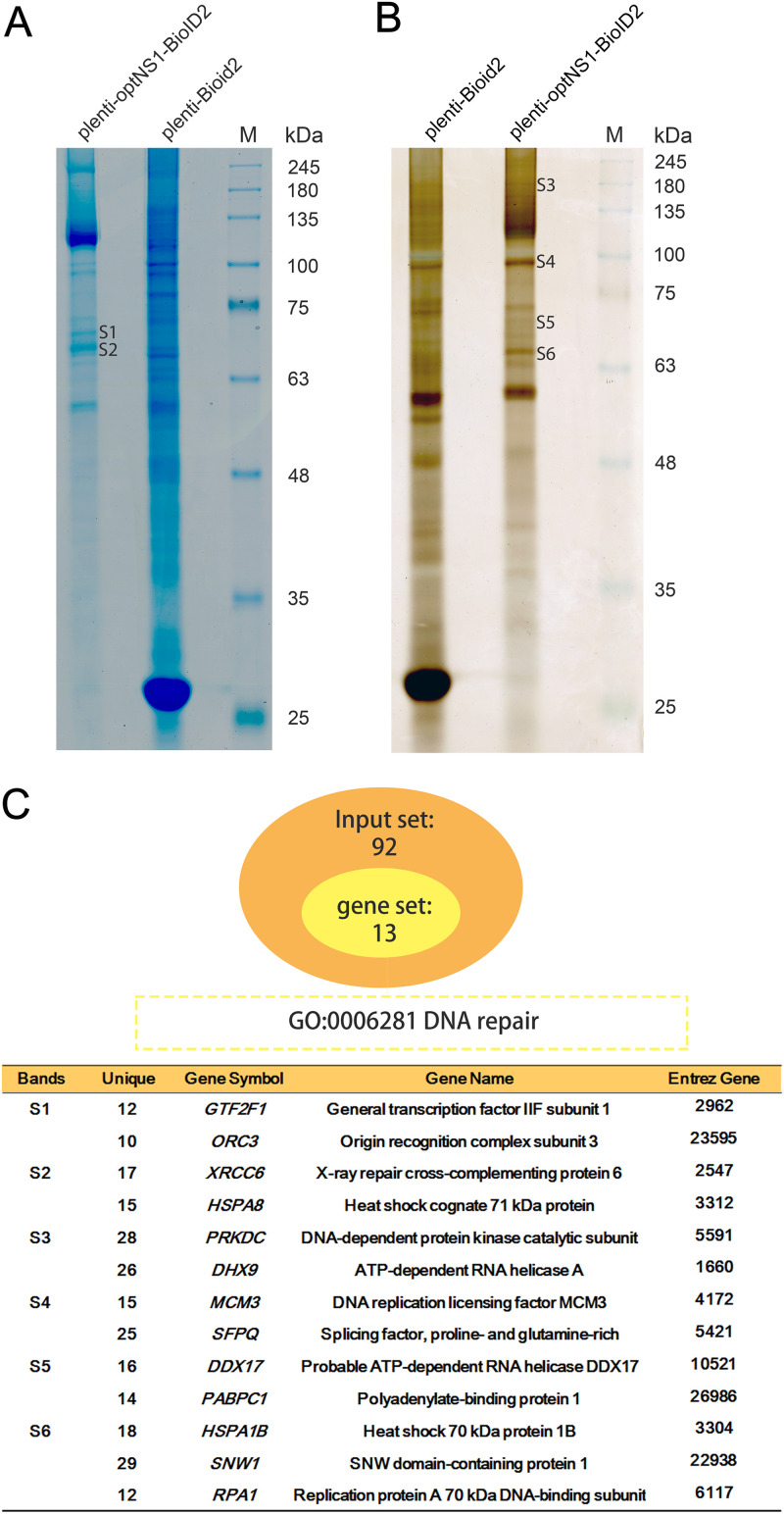
Identification of HBoV1 NS1-interacting proteins using BioID2. (A) Coomassie brilliant blue staining of the biotinylated proteins separated by SDS–10% PAGE. Two unique protein bands pulled down by NS1-BioID2-expressing groups are labeled S1 and S2. (B) Silver staining of SDS–10% PAGE of the biotinylated proteins. Four differentially stained bands pulled down in an NS1-BioID2-expressing group, compared to the control group, are labeled S3 to S6. M, molecular weight ladder. (C) A summary of key NS1-interacting host proteins that have a function in DNA repair. The GO analysis was performed by biological processing of NS1-interacting proteins using Metascape (http://metascape.org/), which analyzed proteins of unique peptides above 10 (109). Proteins identified in each band (S1 to S6) of NS1-BIoID2 are shown with respective gene symbols and names.

To biotinylate neighboring NS1-interacting proteins, we expressed a codon-optimized NS1 open reading frame (ORF) fused with BioID2 (optNS1-BioID2) as a bait protein and BioID2 only serving as the control in HEK293 cells. We then performed a pulldown assay using streptavidin beads to acquire biotinylated proteins, which were separated by SDS-PAGE, followed by staining with Coomassie blue or sliver. We found six bands in the NS1-BioID2 lane that were absent or weak in the control counterpart (Fig. 1A and B). The S1 to S6 bands were excised and analyzed by liquid chromatography-tandem mass spectrometry (LC-MS/MS), which reveals ∼1,900 potential NS1-interacting proteins (see Table S1 in the supplemental material).

**FIG 2:**
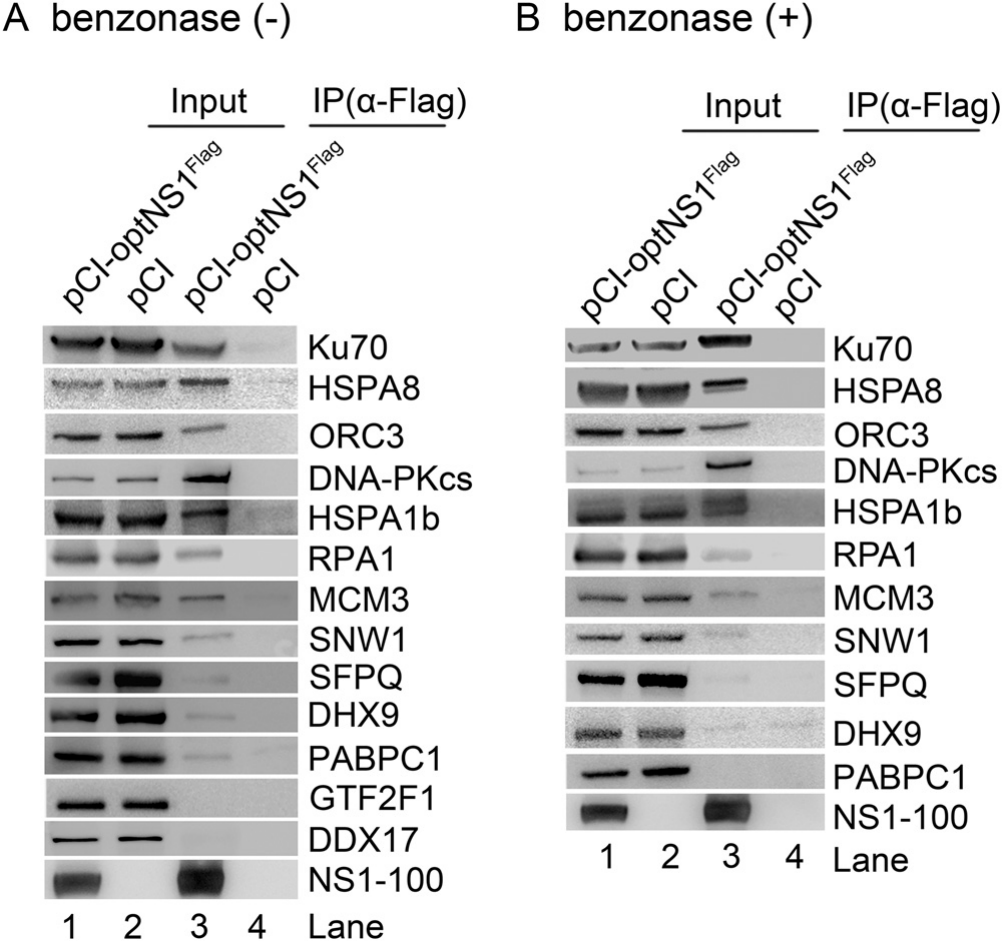
Confirmation of the interaction between the identified DNA repair proteins with HBoV1 NS1 using coimmunoprecipitation (co-IP). HEK293 cells were transfected with pCI-optNS1^Flag^ or pCI (as a vector control). At 48 h posttransfection, immunoprecipitation was performed with anti-Flag-conjugated agarose resin using lysis buffer without (A) or with Benzonase nuclease (B), followed by Western blotting. Proteins detected by respective antibodies are indicated at the right of each image; 10% of the cell lysates was loaded as the input (lanes 1 and 2).

Our goal was to identify host proteins that assist NS1 to replicate HBoV1 DNA. As HBoV1 genome replication is closely associated with the DNA repair and DNA damage response (DDR) pathways in the nucleus (29, 57), we performed gene ontology (GO) analysis of the 92 proteins that have >10 unique peptides identified in LC-MS/MS. The results revealed that 13 proteins have both a nuclear localization and molecular activity associated with DNA replication, DDR, or DNA repair (Fig. 1C). We then performed coimmunoprecipitation (co-IP) to screen the NS1-interacting proteins using NS1-100. To this end, HEK293 cells were transfected with the pCI-optNS1^Flag^ plasmid, while plasmid pCI served as a control. The cell lysates were then incubated with anti-Flag affinity resin for pulldown. As shown in Fig. 2A, 11 proteins (Ku70, HSPA8, ORC3, DNA-PKcs, HSPA1b, RPA1, MCM3, SNW1, SFPQ, DHX9, and PABPC1) were pulled down by NS1-100^Flag^ and anti-Flag beads. To exclude the possibility that the interaction is mediated by DNA or RNA, we added a sufficient amount of Benzonase to the cell lysate to remove any DNA-RNA interaction between the two proteins. This assay identified 8 proteins (Ku70, HSPA8, ORC3, DNA-PKcs, HSPA1b, RPA1, MCM3, and SNW1) that were still obviously pulled down by NS1-100^Flag^ (Fig. 2B).

Collectively, we screened 92 proteins with >10 unique peptides through BioID2 combined mass spectrometry and GO analyses and confirmed 8 proteins, Ku70, HSPA8, ORC3, DNA-PKcs, HSPA1b, RPA1, MCM3, and SNW1, that interact with the NS1 protein in co-IP assays.

### NS1 directly interacts with Ku70, HSPA8, and HSPA1b but not Ku80.

ORC3, RPA1, SNW1, and MCM3 are essential genes for cellular DNA replication (61–65) and, therefore, are nearly impossible to knock down for a function in viral DNA replication without affecting cell viability. DNA-PKcs is a serine/threonine protein kinase comprising a single polypeptide of 450 kDa, whose activation is dependent on the Ku70/Ku80 heterodimer (66, 67). Hence, we focused on Ku70, HSPA8, and HSPA1b for a direct interaction with NS1.

**FIG 3:**
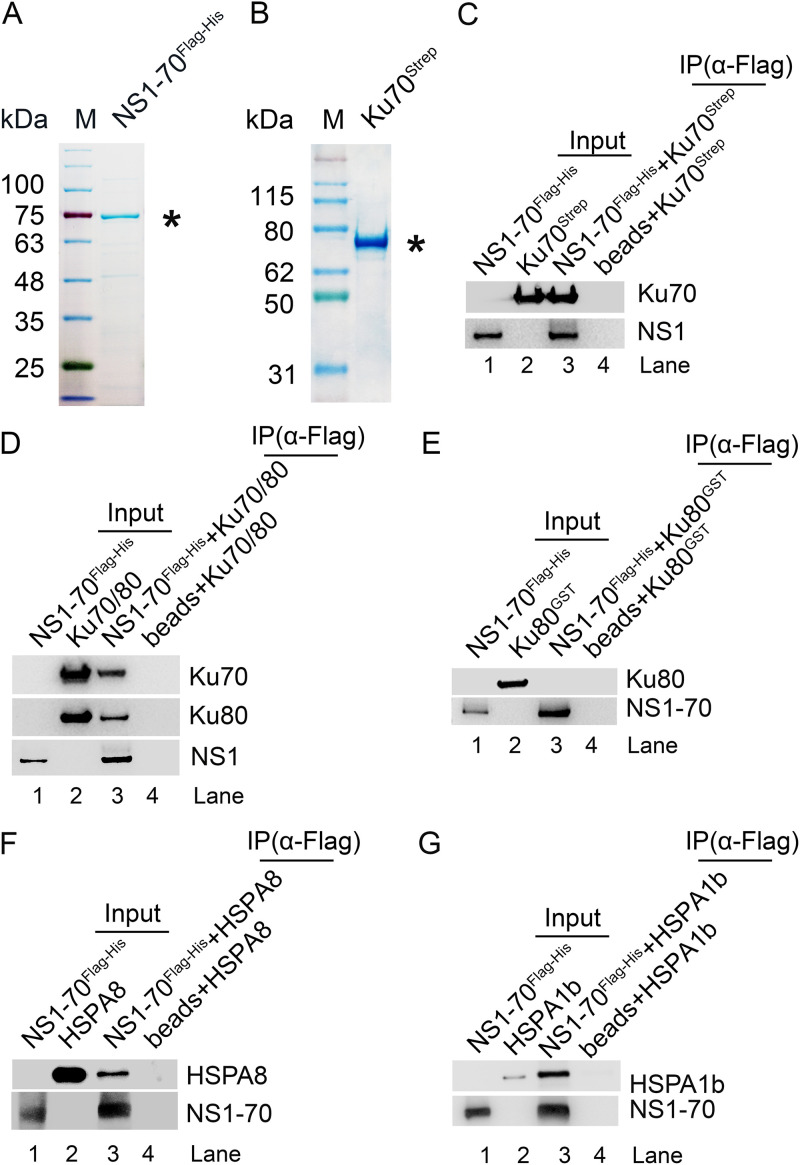
NS1-70 directly interacts with Ku70, HSPA8, and HSPA1b but not Ku80. (A) NS1-70^Flag-His^ purification. NS1-70^Flag-His^ was purified as described in Materials and Methods. The NS1-70^Flag-His^ protein eluted from peaked fractions of the Sephadex G75 column was run on an SDS–10% PAGE gel and then stained with Coomassie brilliant blue dye. (B) Ku70^Strep^ purification. Ku70^Strep^ protein was expressed in BL21(DE3) component cells and purified with Strep-Tactin superflow. The purified Ku70^Strep^ protein was analyzed on an SDS–10% PAGE gel and stained with Coomassie brilliant blue dye. Purified proteins were denoted by asterisks. (C to G) *In vitro* pulldown assay. Two micrograms of purified NS1-70^Flag-His^ was used as bait to pull down 2 μg prey proteins, purified Ku70^Strep^ (C), purified Ku70/Ku80 heterodimer (D), purified Ku80^GST^ (E), purified HSPA8 (F), or HSPA1b (G) using anti-Flag affinity resin. As a negative control, 2 μg prey protein alone (minus bait) was incubated with anti-Flag resin in 400 μl of equilibrium buffer (lane 4 in panels C, D, E, F, and G). The proteins pulled down were analyzed by Western blotting using anti-Ku70 (C and D), anti-GST (E), anti-HSPA8 (F), or anti-HSPA1b (G) and using anti-Flag for NS1-70^Flag-His^ (C to G); ∼0.3 μg of the bait and prey proteins was loaded as inputs. In experiments for panels C and D, we first used 3 U of Benzonase to treat both the bait and prey protein at room temperature for 15 min to remove any DNA/RNA contaminations that may mediate the interaction.

NS1 contains three functional domains, including the N-terminal DNA-binding/endonuclease domain, a helicase domain in the middle, and the C-terminal transcription activation domain, in which the endonuclease and helicase domains are essential to viral DNA replication (28, 68, 69). We noted that it was difficult to purify the large mass of the NS1 protein (NS1-100) in bacteria; thus, we chose to purify the short isoform of NS1, i.e., the NS1-70 protein, which contains the endonuclease and helicase domains and is sufficient to induce a DDR (57) for an assessment of its interaction with Ku70. Recombinant NS1-70^Flag-His^ and Ku70^Strep^ were successfully purified from Escherichia coli (Fig. 3A and B). Purified NS1-70^Flag-His^ pulled down not only purified Ku70^Strep^ (Fig. 3C) but also the purified Ku70/Ku80 heterodimer (Fig. 3D) in the presence of nuclease (Benzonase). Importantly, purified NS1-70^Flag-His^ did not pull down purified Ku80^GST^ (Fig. 3E).

Two molecular chaperones, HSPA8 and HSPA1b, were identified. Molecular chaperones play a pivotal role in the protein quality control system, including correct folding and refolding of proteins and controlling the targeting of proteins for subsequent degradation (70, 71). Our results showed that purified NS1-70^Flag-His^ pulled down purified HSPA8 or HSPA1b (Fig. 3F and G).

Collectively, these results indicate NS1 interacts with the Ku70/Ku80 heterodimer through a direct interaction with Ku70 and that NS1 directly interacts with molecular chaperones HSPA8 and HSPA1b.

### Knockdown of Ku70, but not HSPA8 or HSPA1b, reduced viral DNA replication efficiency in HEK293 cells.

**FIG 4:**
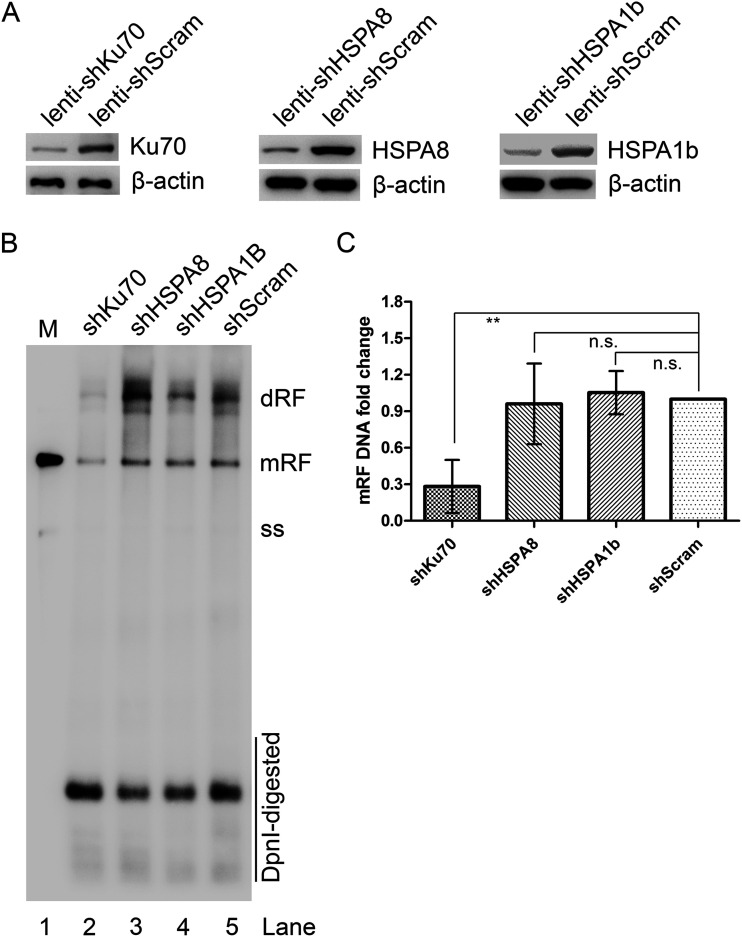
Knockdown of NS1-interacting protein Ku70, but not HSPA8 or HSPA1b, reduced HBoV1 virus replication in HEK 293 cells transfected with pIHBoV1. (A) Knockdown of NS1-interacting proteins in HEK293 cells. HEK293 cells were transduced with lentivirus expressing gene-specific shRNA or scramble shRNA (shScram) as indicated. Cell lysates were identified by Western blotting with respective antibodies (upper) or anti-β-actin antibody (lower). (B and C) Southern blotting. HEK293 cells expressing shRNAs as indicated were transfected with the HBoV1 infectious clone pIHBoV1 (lanes 2 to 5). shScram-expressing cells were also transfected with pIHBoV1 to serve as a positive control (lane 5). At 48 h posttransfection, cells were collected for Hirt DNA extraction. The Hirt DNA samples were separated on 1% agarose gel, which was then transferred onto a nitrocellulose membrane and probed with a [α-^32^P]dCTP-labeled HBoV1 *NSCap* probe. (B) Double replicative form (dRF), monomer RF (mRF), ssDNA bands, and DpnI-digested DNA are indicated. (C) The DNA bands were quantified with Quantity One software (GE Healthcare), and the quantities are presented as levels relative to the Scramble control. n.s., no significance; **, *P* < 0.01.

To further look into the role of Ku70, HSPA8, and HSPA1b in HBoV1 DNA replication, we designed short hairpin RNA (shRNA) to knock down expression of Ku70, HSPA8, and HSPA1b, respectively, in HEK293 cells using shRNA-expressing lentivirus. Western blotting of stably transduced cells showed >4-fold reduction of each protein expressed compared with the scramble shRNA panel (Fig. 4A). We then transfected the HBoV1 infectious clone plasmid pIHBoV1 into those specific shRNA-expressing cell lines. Low-molecular-weight (Hirt) DNAs extracted from transfected cells were loaded on agarose gel for Southern blotting. The results showed that viral DNA replication in the shKu70-expressing cell group was significantly reduced by at least 3-fold compared to the shScram group, while knockdown of HSPA8 or HSPA1b did not have any significant effect on HBoV1 DNA replication (Fig. 4B and C).

Based on these results, we concluded that Ku70, but neither HSPA8 nor HSPA1b, plays an important role in HBoV1 DNA replication in HEK293 cells.

### NS1 interacts with Ku70 throughout the protein, including with the α/β domain, β-barrel, and C-terminal domains, and with the highest affinity at the C-terminal domain.

**FIG 5:**
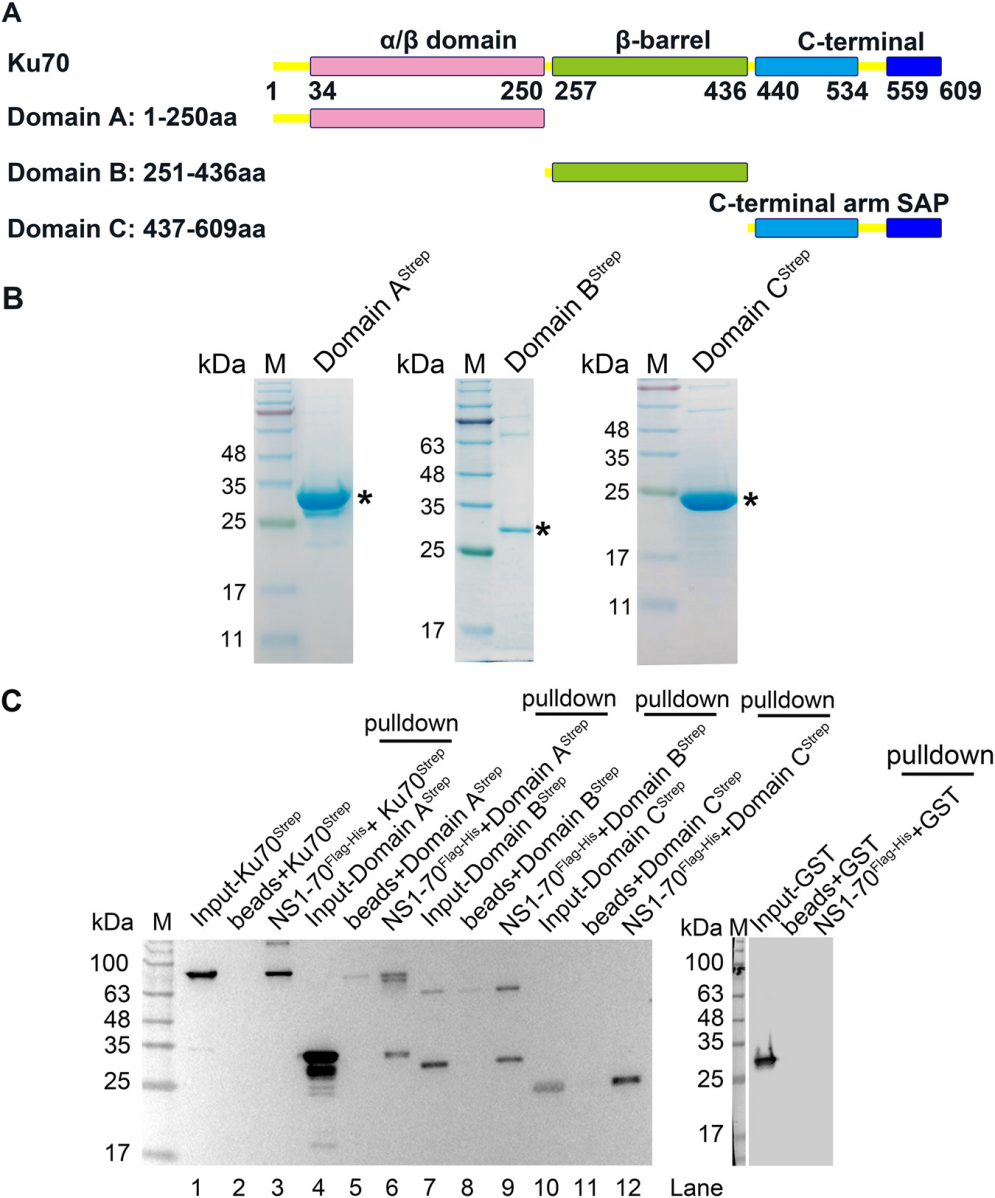
Identification of the Ku70 domain that interacts with NS1-70 using *in vitro* binding assay. (A) A diagram of Ku70 domains. The α/β domain is shown in pink, β-barrel domain in green, and C-terminal domain in blue. Linkers are shown in yellow. (B) Purification of Ku70 domains. All the Ku70 domains were expressed in BL21(DE3) cells and purified using Strep-Tactin agarose. Purified domains were analyzed on SDS–15% PAGE gel and stained with Coomassie brilliant blue dye. Asterisks denote these purified domains. (C) *In vitro* pulldown assay. Two micrograms of purified NS1-70^Flag-His^ was used as bait to pull down 2 μg of prey proteins (Ku70, domain A/B/C, and GST). For Western blotting, we used anti-Strep for Ku70 and its domains and anti-GST for GST protein.

**FIG 6:**
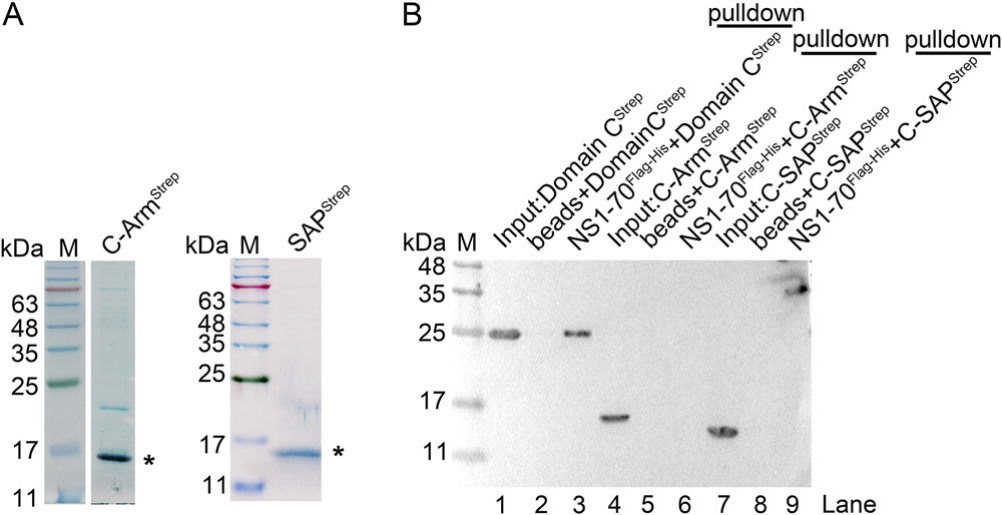
Truncated Ku70 domains C-Arm and SAP did not interact with NS1-70 in an *in vitro* binding assay. (A) Purification of truncated Ku70 domain C. Both C-Arm and SAP domain (see the diagram in Fig. 5A) were expressed in BL21(DE3) *plysS* cells and purified using Strep-Tactin agarose. Purified domains were analyzed on SDS–15% PAGE gel and stained with Coomassie brilliant blue. Asterisks denote the purified domains. (B) *In vitro* binding assay. Two micrograms of purified NS1-70^Flag-His^ was used as bait to pull down 2 μg of prey proteins (C-Arm and SAP domain) using anti-Flag M2 beads. For Western blotting, we used anti-Strep tag II for C-Arm and SAP domains.

Given that NS1 directly interacts with Ku70, we wondered which Ku70 domain plays a role in interacting with NS1. We first expressed and purified each Ku70 domain tagged with Strep-tag II (Fig. 5A), as mapped in previous studies (72–74). Purified proteins were analyzed on SDS-PAGE (Fig. 5B) and used for pulldown assays using NS1-70^Flag-His^, with Ku70 and GST as positive and negative controls, respectively. The results showed that NS1-70^Flag-His^ was able to pull down all three of the Strep-tagged Ku70 domains, A, B, and C (Fig. 5C). We next purified the Strep-tagged terminal arm of Ku70 domain C (C-Arm) and the DNA-binding domain (SAP) of domain C (Fig. 6A). NS1-70^Flag-His^ was used to pull down C-Arm^Strep^ or SAP^Strep^, both of which showed negative results (Fig. 6B). Thus, we next focused on domains A, B, and C for binding kinetics with NS1-70^Flag-His^.

**FIG 7:**
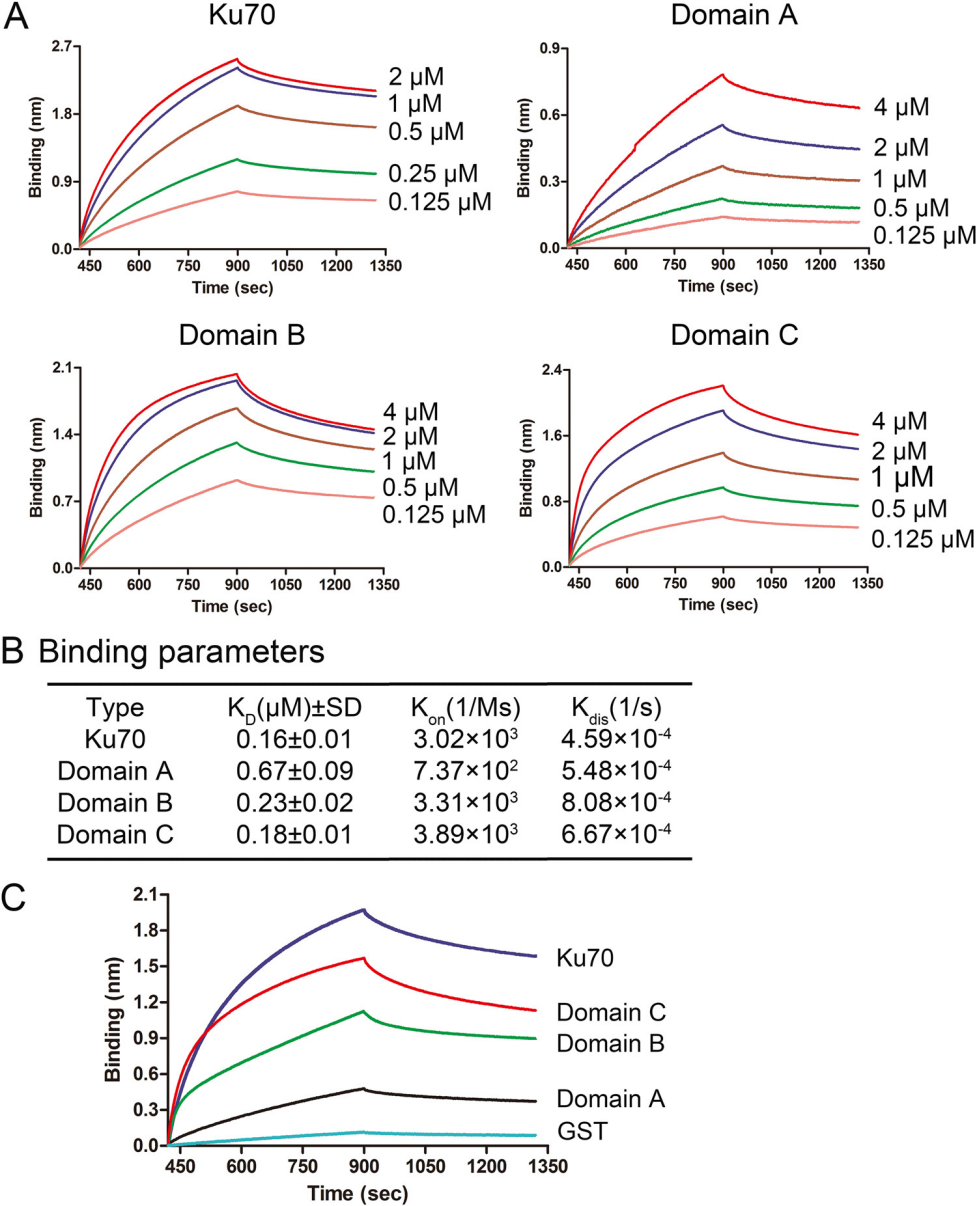
BLI analysis of the binding between NS1-70 with Ku70 and its individual domains. (A) Ni-NTA biosensor kinetic assays. The subtracted kinetics of association and dissociation of NS1-70^Flag-His^ (at 2 μM) with Ku70^Strep^ and its domains (domains A, B, and C) are shown over time at multiple concentrations as indicated. (B) Binding parameters of protein interactions. The *K_D_* value represents affinity constant, *K*_on_ (1/Ms) represents rate of association, and *K*_dis_ (1/s) represents rate of dissociation. The means and standard deviations (SD) from the *K_D_* data were acquired based on at least 3 repeated assays. (C) Comparison of binding affinities. The binding of NS1-70^Flag-His^ (at 2 μM) with Ku70^Strep^ and Strep-tagged domain A, B, or C (at 2 μM) was tested in one experiment, and the subtracted kinetic data of association and dissociation are shown in one plot for comparison. Glutathione *S-*transferase (GST) protein (at 2 μM) was used as a negative protein control.

Biolayer interferometry (BLI) assay has been widely used to determine the kinetics of protein-protein interaction. We then used the BLI assay to quantify the interaction of NS1-70 with Ku70 and its domains. We ran five concentrations of each protein/domain for the binding kinetics with NS1-70 in the BLI assay. The equilibrium dissociation constant (*K_D_*) values, derived from the kinetic constants *K*_on_ (rate of association) and *K*_dis_ (rate of disassociation), which resulted from an average of the binding kinetics parameters using 5 concentrations of Ku70 or its three domains, ranged from 0.16 to 0.67 μM for Ku70 and domains A to C (Fig. 7A and B). This implied that NS1-70 directly binds to domains A, B, and C of Ku70 with a gradually increased *K_D_* value from 0.67 to 0.18 μM, and that the full-length Ku70 has the highest *K_D_* value of 0.16 μM, suggesting a high affinity (75–77). We also compared the binding kinetics of purified Ku70, the three Ku70 domains, and GST (as a negative control) together at 2 μM with 2 μM purified NS1-70. The nonspecific binding of the purified Ku70 and the three Ku70 domains to the NTA biosensors was negligible (data not shown). The result clearly showed domain C has strong binding with NS1-70, which is close to the full-length Ku70, compared to the other two domains (Fig. 7C).

In summary, based on the *in vitro* binding assay and BLI kinetics assay, we verified that NS1-70 directly interacts with Ku70 *in vitro* at a *K_D_* of 0.16 μM throughout all three domains but with the strongest binding to domain C, which has a *K_D_* value of 0.18 μM.

### Ku70 colocalizes with NS1 at the viral replication centers in HBoV1 infectious clone-transfected HEK293 and HBoV1-infected HAE cells.

**FIG 8:**
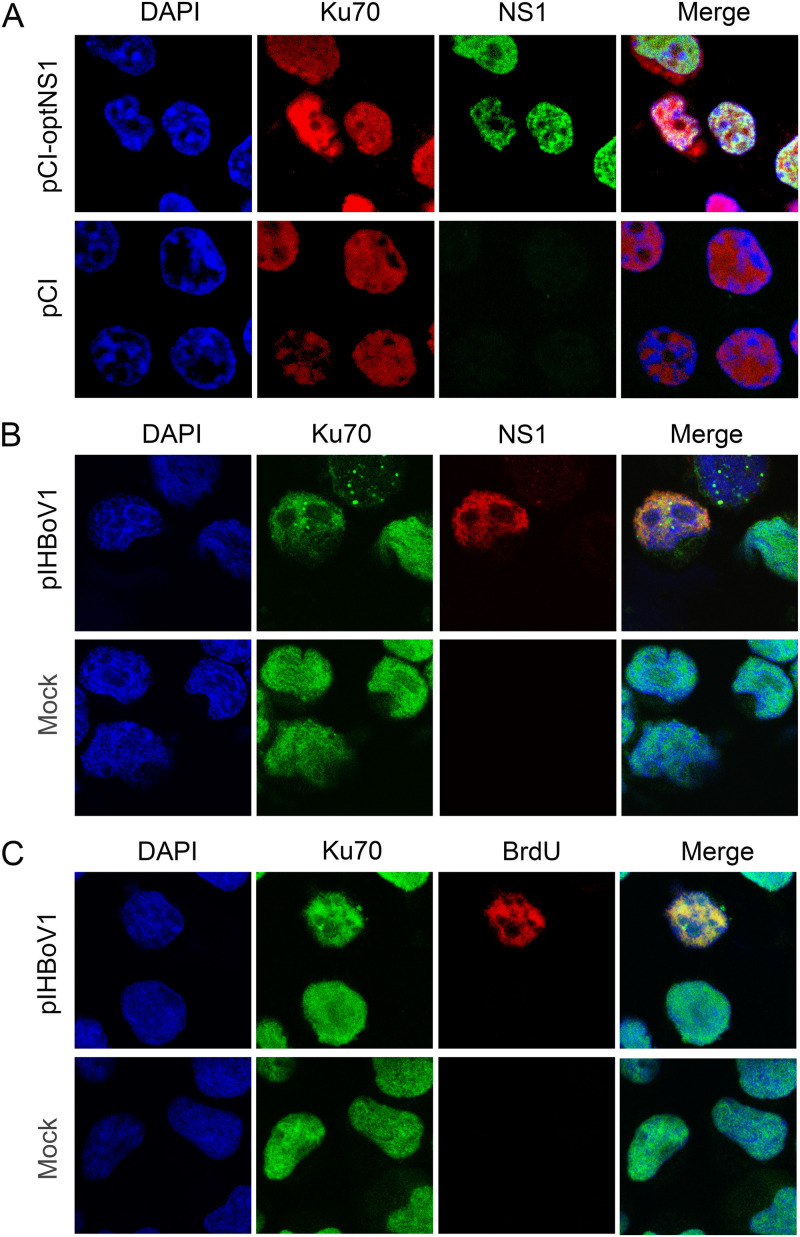
Ku70 is associated with NS1 in transfected HEK293 cells. (A) pCI-optNS1^Flag^-transfected cells. HEK293 cells were transfected with pCI-optNS1^Flag^ or pCI vector. At 48 h posttransfection, the cells were permeabilized and costained with Ku70 antibody (red) and anti-Flag (green). (B and C) pIHBoV1-transfected HEK293 cells. HEK293 cells were transfected with the HBoV1 infectious clone pIHBoV1 or without (Mock). At 48 h posttransfection, the cells were first incubated with 30 μM BrdU and then permeabilized and costained with Ku70 antibody (green) and HBoV1 NS1 antibody (red, B) or anti-BrdU (red, C). Nuclei were stained with DAPI (blue). Images were taken under a Leica TCS SPE confocal microscope at ×63 magnification.

**FIG 9:**
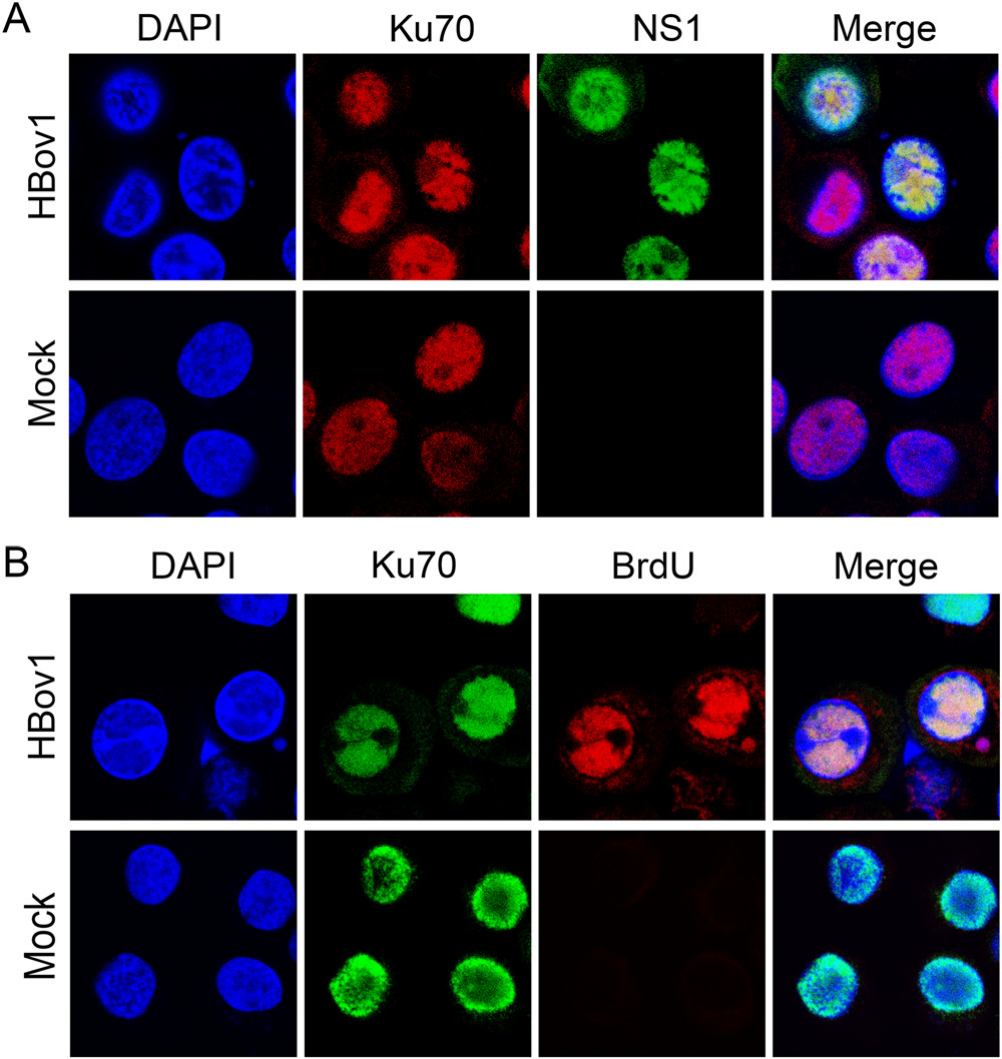
Immunofluorescence assay in HBoV1-infected HAE cells. HAE-ALI cultures were infected with HBoV1 or were mock infected. After 14 days postinfection, the cells were first disassociated from Transwell inserts, incubated with 30 μM BrdU for 30 min, and cytospun onto slides, followed by permeabilization and costaining with anti-Ku70 and anti-HBoV1 NS1 (A) or anti-BrdU (B). Nuclei were stained with DAPI (blue). Images were taken under a Leica TCS SPE confocal microscope at ×63 magnification.

As NS1 is localized in the HBoV1 replication center (29), we examined the colocalization of Ku70 and NS1 in both HEK293 and HAE cells using immunofluorescence analysis (IFA). When we transfected a pCI-optNS1^Flag^ plasmid to HEK293 cells, NS1 was marked with anti-Flag, and Ku70 was detected with its own antibody. We observed that Ku70 and NS1 had a clear colocalization in the nucleus (Fig. 8A). We then transfected the HBoV1 genome plasmid pIHBoV1 to HEK293 cells and added bromodeoxyurdine (BrdU) to the medium before collecting the cells to detect single-stranded DNA (ssDNA) (78), which marks the location of the HBoV1 DNA replication center, which we previously performed during bocaparvovirus MVC infection (36, 42). As shown in Fig. 8B and C, Ku70 colocalized not only with NS1 protein but also with BrdU-labeled replicating viral ssDNA, which is the viral DNA replication center. Similarly, in HBoV1-infected well-differentiated HAE cells, we observed a distinct colocalization between Ku70 and NS1 as well as between Ku70- and BrdU-labeled replicating viral DNA (Fig. 9).

In summary, we verified that Ku70 is colocalized with NS1 during viral DNA replication in both pIHBoV1-transfected HEK293 cells and HBoV1-infected HAE cells, and both are localized in the viral DNA replication center.

### Ku70 plays an important role in viral genome amplification in HAE-ALI.

**FIG 10:**
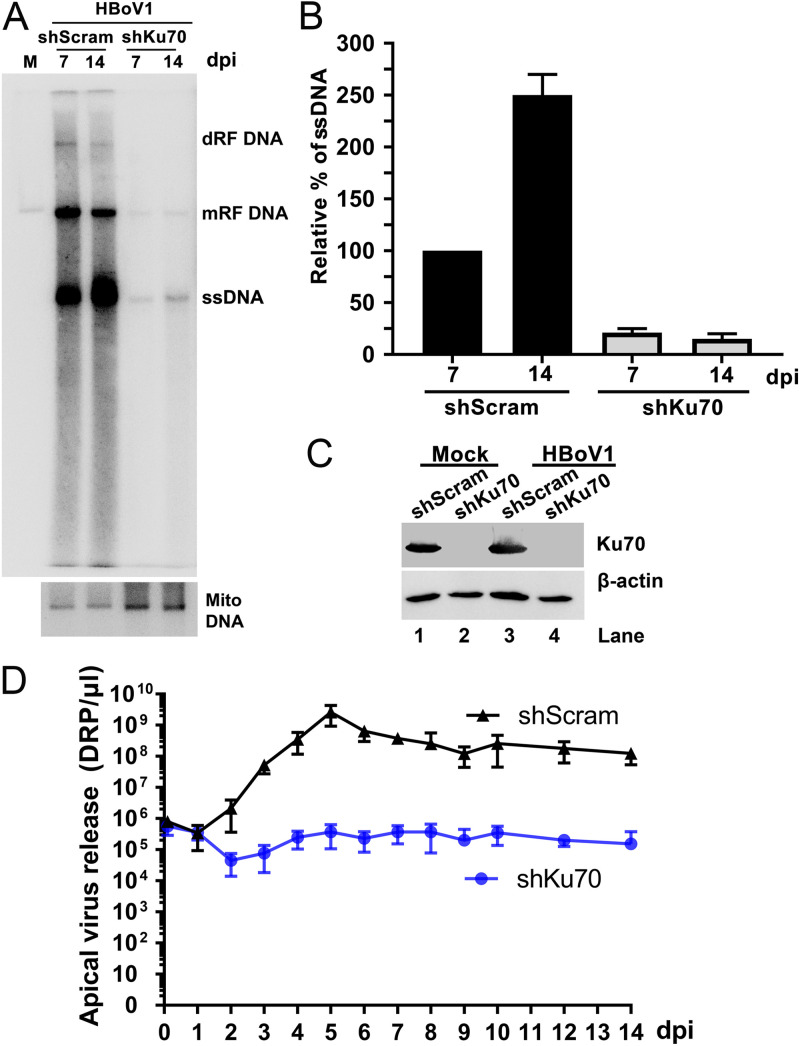
Knockdown of Ku70 expression decreased viral DNA replication in HAE-ALI cultures. (A and B) Southern blotting. Well-differentiated HAE cells of ALI cultures, which had been transduced with shScram or shKu70 lentivirus, as indicated, were infected with HBoV1 at an MOI of ∼100. At 7 and 14 days postinfection (dpi), cells were collected for Hirt DNA extraction, followed by Southern blotting. (A) The blot was probed with an HBoV1 full-length gene probe (top) and the mitochondrial DNA probe (Mito-DNA) (bottom) and imaged on a phosphor screen. dRF and mRF are double and monomer replicative-form DNA, respectively, and ssDNA is single-stranded DNA. (B) The intensity of ssDNA bands was quantified by Quantity One software (Cytiva) and normalized to mitochondrial DNA of each sample. The value representing viral ssDNA in shScram-transduced HAE cells at 7 dpi was set to 100%. Relative values are shown with averages and standard deviations. (C) Ku70 knockdown in HAE-ALI cultures cells. HBoV1-infected or mock-infected shKu70- or shScram-transduced HAE-ALI cultures were lysed for Western blotting with anti-Ku70 antibody. The blots were reprobed for β-actin. (D) Apical virus release. Apical virus release from shKu70- or shScram-transduced HAE-ALI cultures were collected over the course of 14 days. At the indicated days, the apical virus was collected in 100 μl of d-PBS and quantified using quantitative PCR. Values represents means ± standard deviations of the virus collected from 3 infected ALI cultures.

We finally verified the Ku70 function in HBoV1 replication of HAE-ALI cultures. We first transduced shKu70-expressing lentivirus to dividing HAE cells to acquire stable knockdown cells and then infected the cells with HBoV1 when they were well differentiated (79). At 7 or 14 days postinfection (dpi), we extracted Hirt DNA from collected cells for Southern blotting, and the result showed that viral DNA replication in the shKu70-expressing cell group was reduced significantly by ∼5-fold and ∼15-fold at 7 dpi and 14 dpi, respectively, compared to the shScram group (Fig. 10A and B). The shKu70 knockdown efficiency by HBoV1-infected HAE-ALI was confirmed by Western blotting (Fig. 10C). The decrease of virus replication in shKu70-expressing HAE-ALI cultures (HBoV1 infected) was further supported by a reduction of >2 log_10_ in apically released virus during 3 to 14 dpi compared with the shScram-treated HAE-ALI (Fig. 10D). At 5 dpi, apically released virus reached the highest peak of 2.6 × 10^9^ DNase digestion-resistant particles (DRP)/μl in shScram-treated HAE-ALI, whereas there were only 3.7 × 10^5^ DRP/μl of apical virus released from shKu70-expressing HAE-ALI.

**FIG 11:**
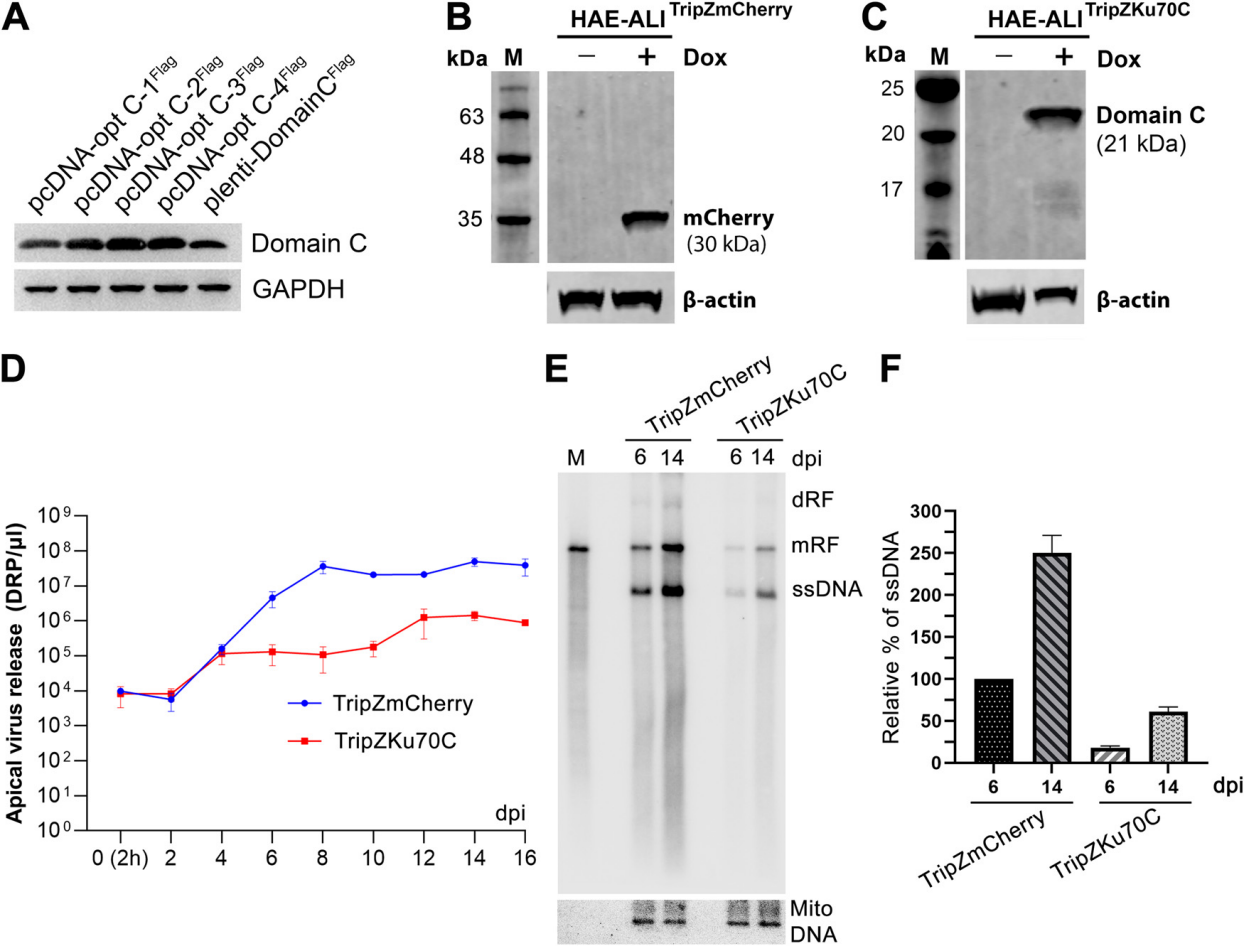
Overexpression of domain C of Ku70 in HAE-ALI significantly decreases apically released virus and viral DNA replication. (A) Codon optimization. HEK293 cells were transfected with four codon-optimized and Flag-tagged Ku domain C cloned plasmids. At 2 days posttransfection, the cells were collected and lysed for Western blotting using anti-Flag. GAPDH was probed as a loading control. (B and C) Induction of mCherry and Ku domain C expression in HAE-ALI. Lentivirus TripZKu70C and TripZmCherry transduced airway epithelial cells were polarized for 4 weeks, and 2 μg/ml doxycycline was added to the basolateral chamber of the ALI cultures. At 1 day posttreatment, the cells were analyzed for expression of mCherry (B) or Ku domain C (C) by Western blotting using anti-Flag. β-Actin was reprobed as a loading control. (D) Apical virus release. Domain C- or mCherry-expressing HAE-ALI cultures were infected with HBoV1 at an MOI of 100 DRP/cell. Apical virus release from infected HAE-ALI cultures was collected over a course of 16 days. At the indicated days, the apical virus was collected in 100 μl of d-PBS and quantified for DRP using qPCR. Values represent means ± standard deviations from the virus collected from 3 infected ALI cultures. (E and F) Southern blotting. At 6 and 14 days postinfection (dpi), cells were collected for Hirt DNA extraction, followed by Southern blotting. (E) The blot was probed with an HBoV1 DNA probe and the mitochondrial DNA probe (mito-DNA), respectively. (F) The intensity of ssDNA bands was quantified by Quantity One software and normalized to mito-DNA of each sample. The value representing viral ssDNA in mCherry expressing (HBoV1-infected) HAE-ALI at 6 dpi was set up to 100%. Relative values are shown with averages and standard deviations.

To further prove the function of the interaction between Ku70 and NS1 in HBoV1 replication, we applied a dominant-negative strategy to induce overexpression of domain C in infected HAE-ALI. To this end, four codon-optimized open reading frames (ORFs) of Ku70 domain C were synthesized and cloned in pcDNA3.1. Transfection of the four Ku70 domain C-expressing plasmids showed domain C-3 and -4 were expressed at a higher level than the wild-type control (Fig. 11A). We next cloned the domain C-3 ORF into an inducible lentiviral vector, TripZ (80). Proliferating airway epithelial cells were transduced with TripZKu70C and a control TripZmCherry lentivirus, respectively. After polarization for 4 weeks, doxycycline was added to the basolateral chamber of the HAE-ALI cultures 1 day prior to HBoV1 infection, and mCherry (control) or domain C expression was induced the next day (Fig. 11B and C). In the time course of 16 days, apical washes were collected for detection of virus release. The result started at 6 dpi with the domain C-expressing HAE-ALI producing 1 to 2 log less virus from the apical side than from the mCherry-expressing HAE-ALI (Fig. 11D). Hirt DNA was prepared at 6 and 16 dpi, respectively, from mCherry- or Ku70C-expressing HAE-ALI and analyzed for viral DNA replication using Southern blotting. The result confirmed that Ku70 domain C-expressing HAE-ALI had decreased viral ssDNA >4 times less than the mCherry-expressing HAE-ALI at both 6 and 16 dpi (Fig. 11E and F).

Taking all these results together, Ku70 plays an important role in HBoV1 replication in HAE-ALI cultures, which is likely through the interaction between Ku70 and NS1.

## DISCUSSION

Viruses have evolved sophisticated strategies to redirect the DNA damage machinery for viral DNA replication through selectively activating or suppressing components of the DDR. Like other small DNA viruses, parvoviruses express only one large nonstructural protein that is essential to viral DNA replication, namely, NS1 or Rep78/68. Therefore, they must exploit cellular DNA damage/repair signaling pathways for viral DNA replication. Two members of the Y-family of DNA repair polymerases, Pol η and Pol κ, have been identified with involvement in HBoV1 genome amplification (29). In this study, we identified a direct interaction of the viral NS1 with Ku70 of the DNA-PK complex, which has a high *K_D_* value of 0.16 μM. Knockdown of Ku70 in HEK293 cells or HAE-ALI significantly decreases viral DNA replication, and overexpression of the key NS1-interacting domain C of Ku70 in HAE-ALI drastically decreased virus replication. Thus, our results explain in part the mechanism underlying how the Ku complex localizes to the viral DNA replication centers, which is likely mediated by the direct interaction between NS1 and Ku70.

In response to AAV2 and AdV coinfection, DNA-PKcs is the primary mediator of the DDR. Ku70, Ku80, and DNA-PKcs, which compose the DNA-PK complex, localize to the AAV2 replication centers (50). An affinity-tagged Rep protein was used to purify cellular protein complexes that were associated with Rep78/52 in cells infected with AAV and AdV or HSV, and the Ku70/Ku80 heterodimer was found in the Rep-associated complex and colocalized in the AAV replication centers (50, 81). In addition to the Rep interaction with Ku70/Ku80 helicase, *in vitro* DNA replication assays demonstrated that Ku70/Ku80 helicase activity could substitute for the minichromosome maintenance protein complex (MCM) to promote strand displacement synthesis (82). Evidence has shown that Ku70/Ku80 and DNA-PK are involved in processes for converting linear input rAAV genomes into circular episomes and concatemers (83–85). Therefore, the Ku/DNA-PK complex plays an important role in AAV2 DNA replication and rAAV2 transduction that also involves a step of DNA repair to form the rAAV episome. Interestingly, the NS1 protein of MVM, B19V, and MVC did not induce a DDR when they were expressed alone (36, 55, 86), suggesting that these NS1 proteins do not interact with Ku70 or other DDR sensor factors.

The Ku heterodimer is composed of two subunits of Ku70 and Ku80, each of which contributes to a central DNA binding core; this core binds to the ends of double-stranded DNA (dsDNA) with high affinity (87) and is required for recruitment of DNA-PKcs to dsDNA breaks *in vivo* (88). During *in vitro* DNA replication of AAV2 DNA, Ku, which interacts with AAV2 Rep78 (82), promotes strand displacement synthesis by acting as a replicative helicase in the absence of the MCM complex (82). Both the Ku and MCM complexes have similar 3′→5′ DNA helicase activities, and both have a preference for replication fork substrates (89, 90). The general mechanism underlying parvoviral DNA replication involves binding NS1 or Rep78 to the NS1- or Rep-binding element, unwinding of the terminus (91, 92), and nicking the top strand of the 5′→3′ orientation at a terminal resolution site (*trs*) (30, 93). Once a nascent replication fork is established, MCM can load onto the strand and unwind the rest of the hairpin to form the hairpin primer required for loading of DNA polymerase and strand displacement synthesis (43, 82, 94). However, in nondividing HAE cells, the cellular DNA replication machinery is shut down (29) and no MCM is expressed, which could explain the more drastic effect of Ku70 knockout on HBoV1 genome amplification in HAE-ALI (Fig. 10). Nevertheless, we still do not understand the requirement of the activation of DNA-PKcs for efficient HBoV1 replication (29).

In the 92 proteins that have >10 peptide reads in the mass spectrometry data of the NS1-BioID2-associated complex, 8 proteins (Ku70, HSPA8, ORC3, DNA-PKcs, HSPA1b, RPA1, MCM3, and SNW1) were shown to interact with NS1 in the pulldown assay. ORC3 is required for the cell cycle and is involved in DNA replication and DNA origin binding activities (61); RPA1 is essential for host DNA replication (62); SNW1 is an essential component of the spliceosome (63, 64); and MCM3 is a component of the MCM complex that is a replicative helicase essential for cellular DNA replication initiation and elongation (65). All three of these protein-coding genes are nearly impossible to knock down for their function in viral DNA replication without affecting cell survival. The RPA heterotrimer has been shown to functionally interact with protoparvovirus MVM NS1 in an *in vitro* viral DNA replication assay (95); thus, the interaction between RPA1 and HBoV1 NS1 warrants further investigation using the *in vitro* viral DNA replication assay. Both HSPA8 and HSPA1b are members of the heat shock protein 70 (HSP70) family and directly interact with NS1. We observed expression of HBoV1 NS1 relocated both HSPA8 and HSPA1b from the cytoplasm to the nucleus with NS1 (data not shown), similar to the redistribution of HSP70 and HSP70 from the cytoplasm into the Kaposi’s sarcoma-associated herpesvirus (KSHV)-induced replication centers in the nucleus, which is independent of their function in protein stability and maturation (96). Host chaperone has emerged with a critical role in RNA virus replication (97) and DNA virus replication (96, 98). However, knockdown of each did not exhibit a defect in HBoV1 viral DNA replication, suggesting that these members of the HSP70 family of proteins function redundantly and independently of their function as molecular chaperones. We speculate other NS1 interacting proteins, ORC3, MCM3, and SNW1, still remain promising candidates to play a role in HBoV1 replication in addition to RPA1, which warrants further investigation.

In conclusion, we, for the first time, identified that a large nonstructural protein of parvovirus, HBoV1 NS1, directly interacts with Ku70 at a high affinity, with a *K_D_* of 0.16 μM. We propose that NS1 functions as a mediator to recruit/concentrate the viral genome (through its DNA binding/endonuclease domain) to the DNA damage foci where Ku70/Ku80 is already present.

## MATERIALS AND METHODS

### Cell lines and primary cultures. (i) HEK293 cells.

HEK293 cells (number CRL-1573; ATCC, Manassas, VA) and HEK293T cells (number CRL-11268; ATCC) were obtained from the ATCC and cultured at 37°C in a 5% CO_2_ atmosphere in Dulbecco’s modified Eagle’s medium (DMEM) (number SH30022.01; Cytiva Life Science, Marlborough, MA) supplemented with 10% fetal bovine serum (number F0926; MilliporeSigma, St. Louis, MO).

### (ii) HAE-ALI culture.

Primary human airway epithelium (HAE) cells cultured in Transwell inserts of 1.12 cm^2^ (number 3460; Corning, NY) at an air-liquid interface (ALI) were generated using a published method (79). Briefly, cells were isolated from bronchial airways, which were obtained from the Cell Culture Core of the Center for Gene Therapy, University of Iowa, as previously described (26, 29), and were propagated in an airway cell expansion medium (PneumaCult-Ex plus medium; number 05040; StemCell, Vancouver, BC, Canada) for one to two passages. When cells became confluent on a collagen-coated 100-mm flask, they were dissociated and transferred onto Transwell inserts at 1.5 × 10^5^ cells per insert. Both basolateral and apical chambers were supplied with Ex Plus medium in the first 2 to 3 days and then fed with PneumaCult-ALI medium (number 05001; StemCell) in the basolateral chamber. With changing ALI medium every 3 days, after 4 weeks, the transepithelial electrical resistance (TEER) was measured. The cultures that had a TEER of over 1,000 Ω/cm^2^ were used for HBoV1 infection.

### Plasmid constructs. (i) NS1 and BioID2 mammalian expression plasmids.

pCI-optNS1^Flag^ has been described previously (28). pLenti-optBioID2 was constructed by insertion of a BioID2-mycNLS-HA ORF into pLenti-IRES-GFP through AgeI-SalI sites (99). The BioID2 ORF encodes a biotin ligase of *A. aeolicus* with a catalytic site mutation (R40G) (60), which was synthesized based on Addgene plasmid number 74224. pLenti-optNS1-BioID2 was constructed by insertion of an optimized HBoV1 NS1-100 (57) at the N terminus of the BioID2 ORF through BamHI and AgeI sites.

### (ii) pLKO constructs.

Lentiviral vector pLKO.1 with a fluorescent reporter, mCherry, was used to clone shRNA hairpin sequences between the AgeI and EcoRI sites (29). pLKO.1-mCherry containing a scramble shRNA sequence was used as a control (shScram) (29). The following shRNAs were obtained from MilliporeSigma for knockdown of Ku70, HSPA8, and HSPA1b: shKu70 (5′-CCG GGA TGA GTC ATA AGA GGA TCA TCT CGA GAT GAT CCT CTT ATG ACT CAT CTT TTT G-3′); shHSPA8 (5′-CCG GGC CCA AGG TCC AAG TAG AAT ACT CGA GTA TTC TAC TTG GAC CTT GGG CTT TTT TG-3′); and shHSPA1b (5′-CCG GCA TCG ACT TCT ACA CGT CCA TCT CGA GAT GGA CGT GTA GAA GTC GAT GTT TTT TG-3′). shScram was used as an shRNA control (5′-CCG GCC TAA GGT TAA GTC GCC CTC GCT CGA GCG AGG GCG ACT TAA CCT GTT TTT G-3′).

### (iii) pTripZ plasmids.

Four plasmids of pcDNA3.1-opt-Ku70 domain C-1/2/3/4, cloned with four codon-optimized C domains tagged with Flag through BamHI and MluI, were purchased from General Biol (Anhui, China). The pTripZKu70C and pTripZmCherry vectors were constructed by inserting C-terminally Flag-tagged opt-Ku70 domain C-3 and mCherry ORFs, respectively, into pTripZ-NS1-Strep-Flag, which has been described previously (80), via the AgeI and MluI restriction sites.

### (iv) Bacterial expression plasmids.

pET30a-NS1-70^His^ and pET30a-Ku70^Strep^ were constructed by inserting NS1-70 open reading frame (ORF) or Ku70 ORF into the pET30a(+) vector (number 69909; MilliporeSigma) between NdeI and XhoI sites. The Ku70 domains expressing pET30a vectors were constructed by inserting Ku70 amino acid 1 to 250, 251 to 440, 437 to 609, 437 to 534, and 535 to 609 coding sequences between the NdeI and XhoI sites for domains A, B, C, C-Arm, and SAP. All constructs expressed the Strep II tag at the C terminus of the target proteins.

### Plasmid DNA transfection.

HEK293 cells and HEK293T cells were transfected by using the PEImax transfection reagent (number 24765-2; Polyscience, Inc., Warrington, PA). The total transfection amounts of plasmid DNA were kept constant at 2 μg per well of 6-well plates, 4 μg per 60-mm dish, and 15 μg per 150-mm dish.

### Viruses, infection, and quantification of apical virus release.

We followed a published method to perform virus collection and infection (29). HAE-ALI cultures were infected with HBoV1 virions at a multiplicity of infection (MOI) of 100 DNase digestion-resistant particles (DRP)/cell as previously described (29). Briefly, viruses were diluted to 100 μl with Dulbecco’s phosphate-buffered saline (d-PBS; number 21-031-CV; Corning). This diluent was incubated at the apical chamber for 1 h, and then the chamber was washed twice with d-PBS. At various time points, apical washes were collected from the apical chamber of HBoV1-infected HAE-ALI cultures by addition of 300 μl d-PBS for 1 h and stored at 4°C for qPCR quantification as described previously (25, 79).

### Lentivirus production and transduction.

We followed the lentivirus production instructions provided by Addgene (http://www.addgene.org/tools/protocols/plko). The concentrated virus was resuspended in cell medium and used for transduction at a multiplicity of infection (MOI) of ∼5 U/cell in HEK293 cells, as described previously (86, 99). To transduce HAE-ALI cultures, we followed a published airway basal cell transduction protocol that we previously used (79). Briefly, we transferred ∼1.5 × 10^5^ proliferating HAE cells onto each Transwell insert in Ex Plus media. After 1 day, the cells were transduced with lentivirus at an MOI of ∼5. At 2 days postransduction, media in the inserts were removed and ALI media were added into the basolateral chamber to induce cell polarization for ∼4 weeks. Transductions of shRNA-expressing lentivirus in HEK293 and HAE-ALI cultures were confirmed by the fluorescence of mCherry expression. For lenti-TripZ-transduced HAE-ALI, doxycycline was added to the basolateral side at 2 μg/μl. At 1 day posttreatment, cells were taken for Western blotting of induced proteins.

### BioID assay.

We performed the BioID assay by following a published method (100, 101). Briefly, HEK293 cells were transfected with plenti-optNS1-BioID2 or plentI-BioID2. At 24 h posttransfection, newly prepared biotin was added into the media to a final concentration of 50 μM, followed by incubation for another 24 h. The cells were washed three times with PBS (pH 7.4) and lysed at room temperature in lysis buffer (50 mM Tris, pH 7.4, 500 mM NaCl, 0.4% SDS, 1 mM dithiothreitol [DTT]) and protease inhibitors (number S8830; MilliporeSigma). Triton X-100 then was added to a final concentration of 2%. After subsequent sonication on ice, prechilled 50 mM Tris buffer (pH 7.4) was added, followed by additional sonication. The cell lysate was centrifuged at 16,500 × *g* at 4°C, and the supernatants were transferred to 100 μl of prewashed streptavidin-conjugated agarose beads, followed by incubation on a rotator at 4°C overnight. The next day, beads were washed twice for 5 min in TBS buffer containing 2% SDS (all subsequent steps were performed at room temperature), which was repeated once with 50 mM Tris buffer (pH 7.4) for 5 min. Finally, the bound proteins were removed from the beads with equal volumes of 2× Laemmli loading buffer and boiled for 5 min before resolving by SDS-PAGE, followed by Coomassie blue or silver staining (SilverQuest silver staining kit; Invitrogen). Differentiated bands from the pLenti-optNS1-BioID2 group were excised and subjected to liquid chromatography-tandem mass spectrometry (LC-MS/MS) analysis at the Taplin Biological Mass Spectrometry Facility, Harvard University.

### Co-IP assay.

For candidate proteins that interacted with the NS1 protein, the pCI-optNS1^Flag^ or pCI vector plasmid was transfected to HEK293 cells cultured in a 145-mm dish, and at 2 days after transfection, the cells were washed twice with cold PBS and lysed with 1 mL lysis buffer (50 mM Tris, pH 8.0, 150 mM NaCl, 1% NP-40, and protease inhibitors) by mixing with discontinuous agitations for 30 min on ice. The cell lysates were treated with or without 250 U of Benzonase (number E8263; Sigma), followed by centrifugation at 12,000 rpm for 10 min at 4°C. The supernatant was then collected, of which 80 μL was boiled in loading buffer as an input control, and the remaining supernatant was incubated with 100 μl of prewashed anti-Flag G1 affinity resin (number L00432; GenScript, Piscataway, NJ) followed by rotation at 4°C overnight. Finally, the beads were washed 3 times with washing buffer (50 mM Tris, pH 8.0, 150 mM NaCl, 1% NP-40, and 1 mM EDTA) for 3 min before mixing with 2× Laemmli loading buffer for Western blotting.

### Protein expression and purification.

The cloned pET30a(+) plasmids were transformed into BL21/DE3 pLysS E. coli bacteria (number L1195; Promega, Madison, WI). A fresh colony harboring the expression plasmid was inoculated into 3 mL of 2× YT medium containing 50 μg/mL kanamycin, followed by overnight shaking at 250 rpm at 37°C. On the 2nd day, the preculture was inoculated in 1 liter of 2× YT medium containing 50 μg/mL kanamycin and shaken at 37°C. At an optical density at 600 nm (OD_600_) of 0.4 to ∼0.6, 1 M isopropyl-β-d-thiogalactopyranoside stock solution was added to the culture to an end concentration of 1 mM, followed by overnight shaking at 16°C. Finally, the bacterial cells were harvested by centrifugation at 5,000 rpm for 15 min (4°C). Recombinant His-tagged proteins were purified as described previously (102, 103).

His-tagged NS1-70 was further run through a Sephadex G75 column on an AKTA purifier (Cytiva) at a flow rate of 0.5 ml/min. The column was washed with TBS buffer (50 mM Tris, pH 7.4, 150 mM NaCl). Peaked fractions were then concentrated using a 50-kDa Amicon Ultra-4 centrifugal filter device (number UFC805008; Merck, Darmstadt, Germany).

Recombinant Strep-tag II-tagged protein-expressing cells were lysed in lysis buffer (50 mM Tris, pH 7.4, 150 mM NaCl, 1 mM EDTA, 1% Triton X-100, 5 mM 3-[(3-cholamidopropyl)-dimethylammonio]-1-propanesulfonate, 1 mM DTT) for 50 min and sonicated for 2 min, followed by centrifugation at 10,000 × *g* for 15 min. The supernatant was mixed with 1 mL TBS prewashed Strept-Tactin Superflow suspension (number 2-1208-010; IBA Life Sciences, Göttingen, Germany), followed by rotation for 2 to ∼3 h at 4°C. The beads then were washed 3 times with 15 ml TBS. Proteins were eluted with 10 ml of elution buffer (100 mM Tris-HCl, pH 8.0, 150 mM NaCl, 1 mM EDTA, 10 mM desthiobiotin).

### *In vitro* pulldown assay.

For pulldown using anti-Flag M2 affinity resin, ∼2 μg of prey protein, the purified Ku70^Strep^ or Strep-tagged Ku70 domains, Ku70/Ku80 heterodimer, HSPA8, or HSPA1b was first mixed with 2 μg bait protein (NS1-70^Flag-His^) in binding buffer (25 mM Tris, pH 7.4, 150 mM NaCl, 1 mM EDTA, 0.1% NP-40) for 3 h. At the same time, 30 μL of anti-Flag M2 resin was blocked with 3% bovine serum albumin (BSA)-PBS for 3 h and incubated with the protein mixture on a rotor for another 3 h at 4°C. Two micrograms of prey protein alone mixed with anti-Flag resin was used as a negative control. The beads then were washed with washing buffer (50 mM Tris·Cl, pH 7.4, 150 mM NaCl, 0.3% NP-40, and 1 mM EDTA) 4 times (each for 12 min). Finally, the bound proteins were eluted by boiling in 2× Laemmli loading buffer and visualized by Western blotting.

Purified Ku70/Ku80 complex (number CT018-H07B; Sino Biological, Wayne, PA), Ku80^GST^ (number H00007520-P01; Abnova), HSPA8 (no. 11329-H07E; Sino Biological), and HSPA1b (number HSP-021; Prospec Bio, Ness-Ziona, Israel) were purchased and used in the study.

### Western blotting.

Western blotting was carried out as previously described (86, 104). Briefly, the cell lysates or other protein samples were resolved on SDS-PAGE gels and transferred onto a nitrocellulose membrane (number 1212590; GVS, Sanford, ME). The transferred membrane then was blocked with 5% nonfat milk and probed with primary and secondary antibodies sequentially. Signals were visualized by enhanced chemiluminescence, and images were developed under a Fuji LAS3000 imaging system (Cytiva).

### *In vivo* DNA replication analysis. (i) Low-molecular-weight (Hirt) DNA extraction.

For pIHBoV1-transfected HEK293 cells, shRNA lentivirus-transduced HEK293 cells were transfected with pIHBoV1. At 48 h posttransfection, the cells were collected. For HAE-ALI cultures, cells in the inserts were first digested with Accutase (number AT104; Innovative Cell Technologies, Inc., San Diego, CA) for 1 h and washed with PBS twice. The dissociated cells then were collected. We followed a previously published method to extract Hirt DNA samples (105). Briefly, the collected cells were washed once with PBS and mixed well with Hirt solution (10 mM Tris·Cl, pH 7.5, 10 mM EDTA, 0.6% SDS) for 15 min. Cell lysates were adjusted to a final NaCl concentration of 1.5 M with 5 M NaCl and incubated at 4°C overnight before being cleared by centrifugation at 14,000 rpm for 20 min. The supernatants were collected and treated with proteinase K at a final concentration of 1 mg/ml for 1 h at 37°C. Hirt DNA samples were further purified by following the DNA gel extraction kit (Qiagen) protocol.

### (ii) Southern blotting.

Southern blotting was performed according to our previously reported methods (30, 105, 106). Briefly, Hirt DNA samples from HEK293 cells were digested with DpnI for 4 h, or samples from HAE cells were directly loaded on a 1% agarose gel and DNA was transferred onto a nitrocellulose membrane. HBoV1 DNA excised from BssHII- and XhoI-digested pIHBoV1 was used as a probe (30). Hybridization signals were captured by using a storage phosphor screen and visualized on a Typhoon FLA 9000 biomolecular imager (GE Healthcare) or an Amersham Typhoon biomolecular imager (Cytiva).

### BrdU incorporation assay.

We followed previously published methods to perform BrdU labeling (36, 42). For pIHBoV1-transfected HEK293 cells, BrdU (MilliporeSigma) was added to the cell culture medium at 30 μM and incubated for ∼30 min prior to dissociation. For infected HAE-ALI cultures, the epithelia on the insert were treated with Accutase (Innovative Cell Technologies, Inc., San Diego, CA) for 30 min. Approximately 1 × 10^5^ cells were resuspended in 1 ml of the PneumaCult-ALI medium (StemCell) with BrdU at 30 μM and incubated for 20 min. The resuspended cells were then cytospun onto slides for immunofluorescence assay as described below.

### IFA.

IFA was performed as previously described (86). Briefly, transfected or infected cells were collected and cytospun onto slides at a speed of 1,800 rpm for 3 min. They were then fixed with 4% paraformaldehyde (PFA) in PBS for 15 min, washed in PBS three times shortly, and permeabilized with 0.5% Triton X-100 for 10 min. Both the 1st and 2nd antibodies were diluted in PBS with 2% FBS. The slides were blocked with 3% BSA-PBS for 1 h, followed by subsequent incubation with 1st antibody, fluorescent dye-conjugated 2nd antibodies, and DAPI (4′,6-diamidino-2-phenylindole) at 2 mg/ml. The cells were then visualized using a Leica TCS SPE confocal microscope at the Confocal Core Facility of the University of Kansas Medical Center.

### BLI assay.

BLI kinetics analysis was performed on the Octet RED96e system (ForteBio/Sartorius, Bohemia, NY) and followed a previously reported method (107). Briefly, the biosensors were first hydrated with kinetic buffer (PBS, 0.02% Tween 20, 0.1% BSA, 0.05% sodium azide) for 10 min, and then the purified NS1-70 protein (2 μM) in kinetic buffer was immobilized on nickel-nitrilotriacetic acid (Ni-NTA) biosensors (number 18-5101; Sartorius, Goettingen, Germany). The biosensors were then dipped into analyte-containing buffer, which contains purified Ku70, Ku70 domains, and glutathione *S*-transferase (GST), respectively. Finally, the biosensors were dipped into the kinetic buffer to finish a dissociation step.

To analyze the binding data, we first selected a reference well that only contains the kinetic buffer to subtract the nonspecific binding of NS1 with the kinetic buffer, and then we used the global fitting model to analyze the subtracted data. All the kinetic data, including the equilibrium dissociation constant, *K_D_*, rate of association, *K*_on_, and rate of disassociation, *K*_dis_, were analyzed using Octet data analysis 4.0 software. The *K_D_* ± standard devastation values were determined by repeating experiments at least three times.

### Antibodies used in the study. (i) Primary antibodies.

Rat anti-HBoV1 NS1C antibody was produced previously (108). The following primary antibodies were purchased: mouse anti-Flag (number 200-301-B13) from Rockland (Limerick, PA); rabbit anti-Strep-tag II (number ab183907) from Abcam (Waltham, MA); mouse anti-β-actin (number A5441) from MilliporeSigma; mouse anti-Ku70 (number SC-17789) and mouse anti-GST (number SC-138) from Santa Cruz (Dallas, TX); rabbit anti-Ku70 (number A7330), rabbit anti-HSPA8 (number A14001), rabbit anti-GTF2F1 (number A2489), rabbit anti-ORC3 (number A15415), rabbit anti-DNA-PKcs (number A1419), rabbit anti-DHX9 (number A4563), rabbit anti-SNW1 (number A14580), rabbit anti-PABPC1 (number A14872), rabbit anti-DDX17 (number A17078), rabbit anti-RPA1 (number A0990), and rabbit anti-MCM3 (number A1060) from Abclonal (Woburn, MA); rabbit anti-SFPQ (number A301-321A-T) from Bethyl (Montgomery, TX); rabbit anti-HSPA1b (number A59434-020) from EpiGentek (Farmingdale, NY); rat anti-HSPA8 (number NBP1-97868) from Novus Bio (Centennial, CO); anti-BrdU (clone B44) from BD Biosciences (San Jose, CA); and anti-GAPDH (number 2118S) from Cell Signaling (Danvers, MA).

### (ii) Secondary antibodies.

Horseradish peroxidase (HRP)-conjugated anti-mouse IgG (number A4416) and HRP-conjugated anti-rabbit IgG (number A0545) were purchased from MilliporeSigma; HRP-conjugated anti-rat IgG (number 112-035-003), fluorescein isothiocyanate (FITC)- and rhodamine-conjugated anti-mouse IgG (number 715-095-151 and number 715-295-151), Alexa Fluor 594-conjugated anti-rabbit IgG (number 711-585-152), and Alexa Fluor 488-conjugated anti-rat (number 712-545-153) were purchased from Jackson Immuno Research Inc. (West Grove, PA).

### Statistical analysis.

Statistical analysis was performed by using GraphPad Prism version 8.0. Error bars represent means and standard deviations. Statistical significance *P* values were determined by using Student's *t* test. *P* values of <0.0001 (****), <0.001 (***), <0.01 (**), and <0.05 (*) were regarded as statistically significant and n.s. as statistically not significant.
